# Supplementary material for: Effects of a forefoot strengthening protocol on explosive tasks performance and propulsion kinetics in athletes: a single-blind randomized controlled trial
Source: PLoS One. 2025 Jun 2;20(6):e0313979. doi: 10.1371/journal.pone.0313979 (PMC12129235; doi:10.1371/journal.pone.0313979)
Supplement: S2 Fig — (PDF) [file pone.0313979.s002.pdf]

FOREFOOT STRENGTHENING PROTOCOL « ENHANCING THE PROPULSING FOOT »

8 WEEKS
2 sessions / week

1 SUPERVISED SESSION

- EXERCISE 1: FOREFOOT ISO-PUSH + NMES
- EXERCISE 2: 1ST RAY DYNAMIC ISO-HOLD
- EXERCISE 3: FOREFOOT REBOUND JUMPS

1 UNSUPERVISED SESSION

- EXERCISE 1: FOOT BRIDGE ISO-HOLD + NMES
- EXERCISE 2: FOREFOOT ISO WALL PUSH

SUPERVISED SESSION - EXERCISE 1: FOREFOOT ISO-PUSH + NMES

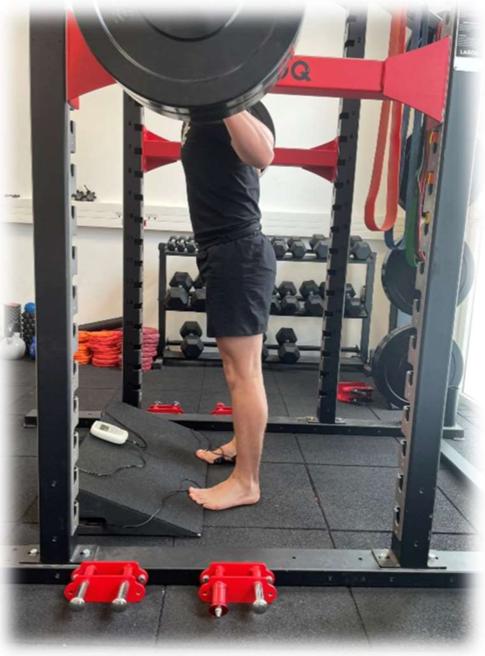

VIDEO

INSTRUCTIONS:

– Maximal isometric MTPj flexion (bilateral)
– MTPj in dorsiflexed position (inclined plate)
– Forward lean movement during the contraction
– NMES surimposed stimulation (under the arch)
– Maximum tolerable NMES intensity

LINK: https://youtube.com/shorts/301AP25dGoU?feature=share

WEEK 1 & 2:	WEEK 3 & 4:	WEEK 5 & 6:	WEEK 7 & 8:
Body over-load: +80%BW	Body over-load: +100%BW	Body over-load: +120%BW	Body over-load: +140%BW
3 x 8 x 8 sec (6"/1'15)	3 x 6 x 8 sec (6"/1'15)	3 x 8 x 4 sec (8"/2'00)	3 x 6 x 4 sec (8"/2'00)
NMES' resistance program (cycle 2)	NMES' resistance program (cycle 2)	NMES' reinforcement program	NMES' reinforcement program
Total iso-push time= 192s	Total iso-push time= 144s	Total iso-push time= 96s	Total iso-push time= 72s

SUPERVISED SESSION – EXERCISE 2: 1ST RAY DYNAMIC ISO-HOLD

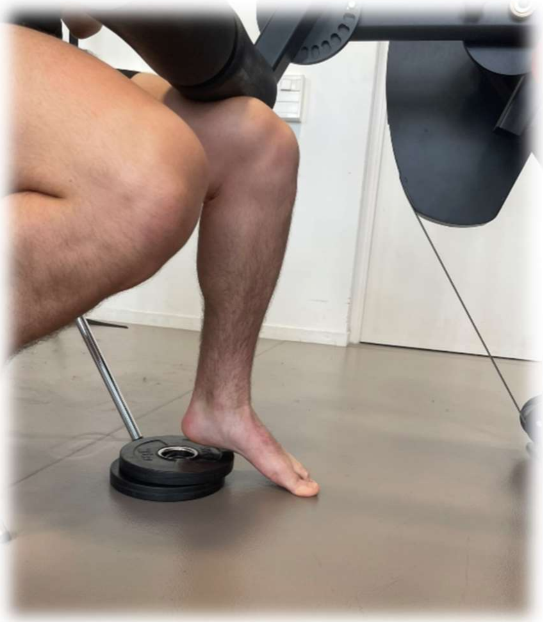

VIDEO

INSTRUCTIONS:

- Maximal isometric 1st ray flexion (unilateral)
  - Separation from the floor of metatarsal' head
  - No interphalangeal joint flexion (you should always see your nails)
- Alternate feet between series

LINK: https://youtube.com/shorts/vbMeNmHMUYE?feature=share

WEEK 1 & 2:	WEEK 3 & 4:	WEEK 5 & 6:	WEEK 7 & 8:
5 sec iso-hold load	5 sec iso-hold load	3 sec iso-hold load	3 sec iso-hold load
3 x 8 x 5 sec (2"/1'15)	4 x 6 x 5 sec (2"/1'15)	3 x 8 x 3 sec (2"/2'00)	4 x 6 x 3 sec (2"/2'00)
Maximal isometric intensity	Maximal isometric intensity	Maximal isometric intensity	Maximal isometric intensity
Total iso-push time= 120s	Total iso-push time= 120s	Total iso-push time= 72s	Total iso-push time= 72s

<u>SUPERVISED SESSION</u> - EXERCISE 3: FOREFOOT REBOUND JUMPS

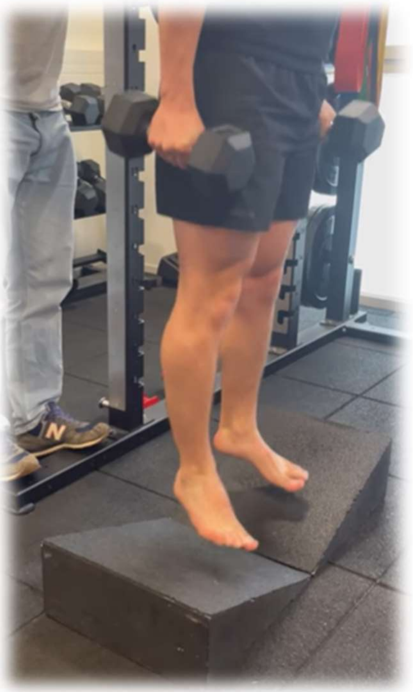

VIDEO

INSTRUCTIONS:

– Vertical foot-ankle rebound jumps (bilateral)
– Forefoot strike with the ground
– Straight leg (minimal knee flexion)
– Minimal ground contact time
– Jump height emphasize

LINK: https://youtube.com/shorts/RehkkkvNJfs?feature=share

WEEK 1 & 2:	WEEK 3 & 4:	WEEK 5 & 6:	WEEK 7 & 8:
Body over-load: +10%BW	Body over-load: +20%BW	Body over-load: +30%BW	Body over-load: +40%BW
5 x 10 sec (1'00)	5 x 10 sec (1'00)	5 x 10 sec (1'00)	5 x 10 sec (1'00)
3 series: on everted inclined plate & 2 series: on inverted inclined plate	3 series: on everted inclined plate & 2 series: on inverted inclined plate	3 series: on everted inclined plate & 2 series: on inverted inclined plate	3 series: on everted inclined plate & 2 series: on inverted inclined plate

UNSUPERVISED SESSION - EXERCISE 1: FOOT BRIDGE ISO-HOLD + NMES

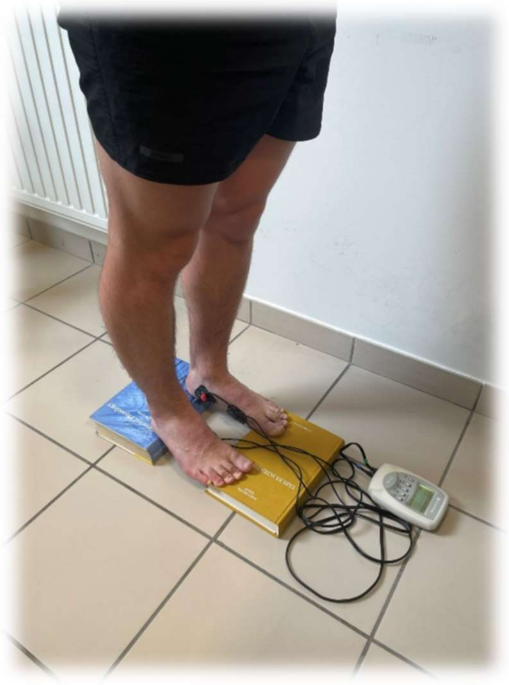

VIDEO

INSTRUCTIONS:

- Forefoot and rearfoot on plates (midfoot unloading)
– Hold the medial arch curvature (bilateral)
– Forward lean movement during the contraction
– NMES surimposed stimulation (under the arch)
– Maximum tolerable NMES intensity

LINK: https://youtube.com/shorts/MdAe0nU0VHY?feature=share

WEEK 1 & 2:
Load: bodyweight

15 min of NMES' resistance program: 8 sec of contraction - 6 sec of rest

Total iso-hold time= 512s

WEEK 3 & 4:
Load: bodyweight

15 min of NMES' resistance program: 8 sec of contraction - 6 sec of rest

Total iso-hold time= 512s

WEEK 5 & 6:
Load: bodyweight

15 min of NMES' reinforcement program: 4 sec of contraction - 8 sec of rest

Total iso-hold time= 225s

WEEK 7 & 8:
Load: bodyweight

15 min of NMES' reinforcement program: 4 sec of contraction - 8 sec of rest

Total iso-hold time= 225s

UNSUPERVISED SESSION - EXERCISE 2: FOREFOOT ISO WALL PUSH

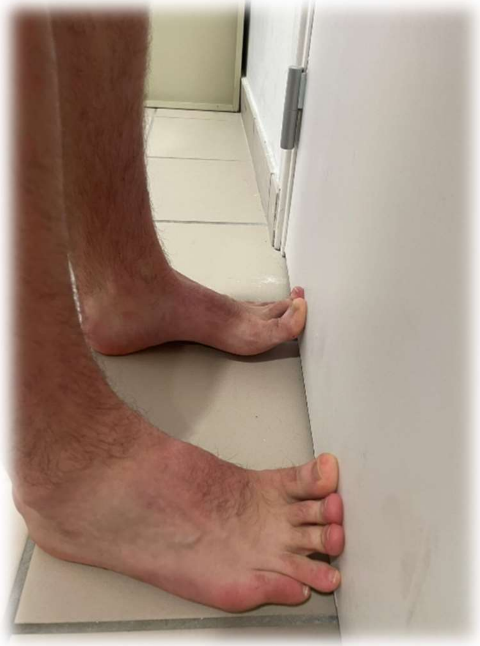

VIDEO

INSTRUCTIONS:

– Maximal isometric MTPj flexion (bilateral)
– MTPj in maximal dorsiflexed position (wall)
– No interphalangeal joint flexion (you should always see your nails)
– Metatarsal' head should move away from wall

LINK: https://youtube.com/shorts/ulSHrZNbwQc?feature=share

WEEK 1 & 2:	WEEK 3 & 4:	WEEK 5 & 6:	WEEK 7 & 8:
Sitting position	Sitting position	Standing position	Standing position
3 x 4 x 5 sec (10"/1'30)	3 x 5 x 5 sec (10"/1'30)	3 x 4 x 5 sec (10"/1'30)	3 x 5 x 5 sec (10"/1'30)
Maximal isometric intensity	Maximal isometric intensity	Maximal isometric intensity	Maximal isometric intensity
Total iso-push time= 60s	Total iso-push time= 75s	Total iso-push time= 60s	Total iso-push time= 75s
