# Supplementary material for: Effects of a forefoot strengthening protocol on explosive tasks performance and propulsion kinetics in athletes: a single-blind randomized controlled trial
Source: PLoS One. 2025 Jun 2;20(6):e0313979. doi: 10.1371/journal.pone.0313979 (PMC12129235; doi:10.1371/journal.pone.0313979)
Supplement: S3 File — (PDF) [file pone.0313979.s003.pdf]

Supplemental Digital Content. Detailed study protocol for sprint acceleration, cutting and jumping kinetics analysis

1 Sprint acceleration task

After an individual warm-up, participants performed sprint accelerations in three different conditions previously described (1,2). During the first condition, “high-acceleration”, the initial start was located 30 cm before the edge of the first force platform. This condition was used to analyze GRF during the early phase of the sprint acceleration (0 to 6 m), during which the magnitude of the average forward acceleration is important (1). The “medium-acceleration” condition was then performed with start located 7 m before the edge of first force platform. This condition was used to analyze the GRF during the mid-acceleration phase (7 to 13 m), during which the magnitude of the average forward acceleration decreases compared to the high-acceleration condition (1). Finally, the “low-acceleration” condition was performed, with a starting point located 30 m before the edge of the first force platform, to analyze the GRF during the final phase of the acceleration (30 to 36 m) when participants approach or reach their maximum running speed (1) (Figure 1). For all conditions, participants were instructed to “sprint as fast as possible” from a three-point crouched start position. As for similar studies on sprint acceleration biomechanics and performance, the trial with the highest performance was analyzed (3,4), i.e. the best 10, 17 and 34-m sprint time assessed using timing gates (Microgate, Bolzano, 113 Italy) set at a height of 1.2 m and placed the beginning of the force platform system. For this best trial, the mean of the GRF components were computed for each valid dominant foot contact. A valid foot contact was defined when the entire foot landed within the boundaries of a single force platform, and checked by smartphone slow motion videos.

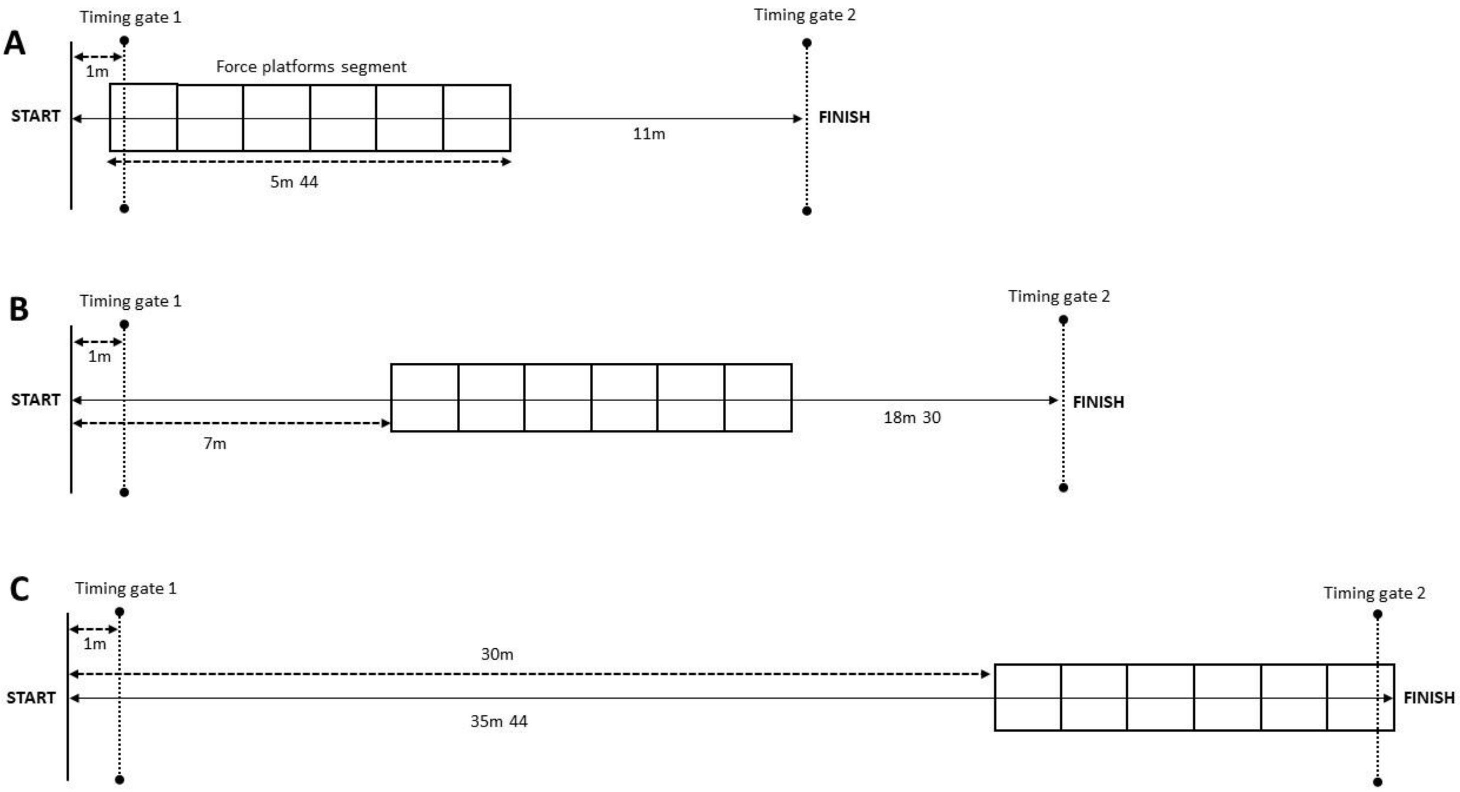

<u>Figure 1</u>: Force platforms setting for A) High-acceleration condition; B) Medium-acceleration condition; C) Low-acceleration condition

GRF signals were filtered using a Butterworth low-pass digital filter at a cutoff frequency of 50 Hz (4). Instantaneous data of vertical (Fz), anteroposterior (Fy) and mediolateral (Fx) and resultant GRF (FTot) were exported and averaged for each contact phase using a custom MATLAB script (Mathworks, USA). Then, numerous discrete GRF variables were calculated based on previous studies (1,2,4) and included: (a) the impulse (integral over time) of the vertical GRF; (b) the net impulse of the horizontal GRF and the impulse of each component of the horizontal GRF calculated separately, i.e. the negative (braking) and positive (propulsive) components; (c) the effective impulse of the vertical GRF i.e. the product of the stance phase duration and the vertical GRF applied in excess of BW; (d) the ratio of forces (RF), i.e the ratio of the step-averaged Fy component divided by the resultant of the step-averaged (FTot); (e) the

contact time defined by the events of foot-strike and toe-off from the raw GRF data (Fz threshold of 20 N) (4).

2 Cutting task

The cutting task was a “90° anticipated” cut performed on the dominant limb. Starting in a staggered position, participants were instructed to run “as fast as possible” for 5 m, make a single complete foot contact within a 90x90 cm area in front of the marker (ensuring their foot was fully in contact with the force platform), and perform a 90° cut before running “as fast as possible” again for 5 m to the finish line. Multiple practice attempts were allowed to reach consistency in the approach speed and trials were repeated if a full foot contact with the force platform was not achieved. The trial with the best performance was used for statistical analysis and considered as the best cutting time using timing gates (Microgate, Bolzano, 113 Italy) placed 2 m before and after the cutting point (Figure 2). The 2-m distance was chosen because it allowed only one step before the cutting movement to increase the acceleration component and reduce the influence of deceleration on the cutting intensity (5).

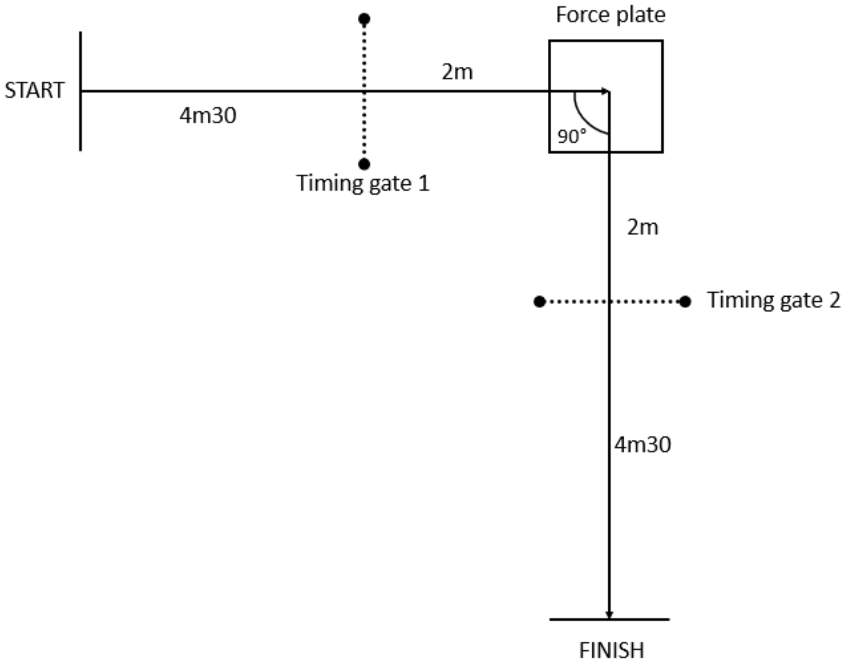

<u>Figure 2</u>: Force platforms setting for the "90° anticipated cut"

GRF signals were filtered using a Butterworth low-pass digital filter at a cutoff frequency of 30 Hz. The braking phase was defined as the time from foot contact onset time at minimal Fz of the mid-support phase, while the propulsive phase was defined as the time from the minimal Fz of the mid-support phase to toe-off (6). Several discrete GRF variables were calculated based on previous studies (6,7) and included for each phase (global, braking, propulsive): (a) the impulse of the vertical, horizontal and medio-lateral GRF; (b) the effective impulse of the vertical GRF; (c) the braking ratio of forces (BRF), i.e the ratio of the stance-averaged Fy component divided by the resultant FTot; (d) the redirection ratio of forces (RRF), i.e the ratio of the stance-averaged Fx component divided by the resultant FTot; and (e) the contact time defined by the events of foot-strike and toe-off from the raw GRF data (Fz threshold of 10N) (6).

3 Jumping tasks

Finally, participants performed jumping tests in three different conditions. The first two were a "vertical and horizontal CMJ" for which participants were instructed to "jump as fast and as high as possible" or to "jump as fast and as far as possible", while constantly keeping their hands on their hips. Verbal cues were standardized following previous recommendations (8) since they can influence CMJ force-time characteristics. The force platforms were zeroed before each trial and the trials with the highest performance (jump height for "vertical CMJ" and the best horizontal peak power for "horizontal CMJ") were used for statistical analysis. GRF signals were filtered using a Butterworth low-pass digital filter at a cutoff frequency of 30 Hz. Instantaneous data of vertical (Fz) and horizontal (Fy) GRF on both feet were summed and COM acceleration was calculated by dividing the GRF data by the athlete's body mass. Then, vertical and horizontal COM velocity and displacement were calculated by integration using the trapezoid approach (9). Movement onset was defined as the moment at which the vertical

force-time curve dropped below a threshold equal to five times the standard deviation of the GRF signal averaged during a 1-second weighing phase (8). Take-off was defined as the moment when the vertical force-time curve dropped below a threshold of 1% BW. The braking phase was defined as the duration from movement onset until the vertical velocity of the COM reached 0 m/s. The propulsive phase was defined as the duration from the end of the braking phase until take-off. From the raw GRF, the following discrete variables (dominant foot) were analyzed for the braking and propulsive phases: (a) the impulse of the vertical and horizontal GRF; and (b) the RF i.e the ratio of the phase-averaged Fy component divided by the resultant of the phase-averaged Fz and Fy components (FTot) for the “horizontal CMJ”. For the global phase the following variables were analyzed: (c) the jump height derived from the impulse-momentum relationship (9), and (d) the peak power derived from the product of the vertical or horizontal velocity and vertical or horizontal GRF.

Finally, the third jumping condition was the FARJ described as a repeated vertical jump to evaluate foot-ankle reactive strength and stretch-shortening cycle capacities (10,11). For this jumping task, participants were instructed to jump “as high as possible” while keeping their lower limbs fully straight, and to push against the ground “as quickly as possible” with only a plantarflexion of the ankle and the MTPj (11) (Figure 3). To avoid hip and knee contribution an observer checked immediately after the trial the amount of hip and knee flexion during the support phase using a slow-motion (240 fps) video. Multiple practice attempts were allowed to familiarize participants with the correct technique. Incorrect jump trials were discarded and repeated. From the eight continuous jumps, the mean of four jumps (excluding the first and last two jumps) was used for statistical analysis. Based on the contact time and flight time measured with an optoelectronic system (Optojump Next, Microgate, Bolzano, 113 Italy) the reactive strength index was calculated as the ratio of jump height (using the flight time method) to contact time (10).

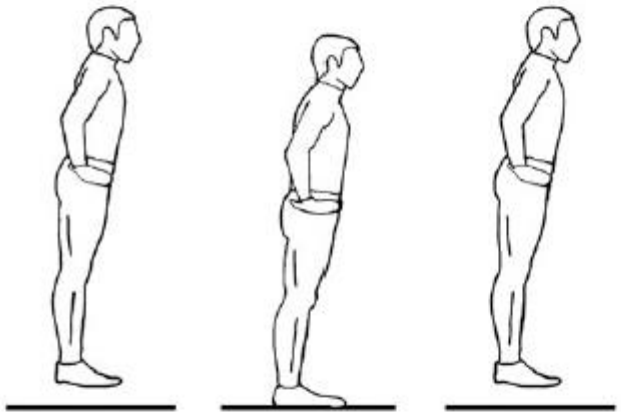

Figure 3: Foot-ankle rebound jump modalities (from Nagahara et al., 2014)

References

1. Schache AG, Lai AKM, Brown NAT, Crossley KM, Pandy MG. Lower-limb joint mechanics during maximum acceleration sprinting. J Exp Biol. 2019 Nov 15;222(22):jeb209460.

2. Rabita G, Dorel S, Slawinski J, Sàez-de-Villarreal E, Couturier A, Samozino P, et al. Sprint mechanics in world-class athletes: a new insight into the limits of human locomotion: Sprint mechanics in elite athletes. Scand J Med Sci Sports. 2015 Oct;25(5):583–94.

3. Nagahara R, Zushi K. Development of maximal speed sprinting performance with changes in vertical, leg and joint stiffness. J Sports Med Phys Fitness. 2017 Dec;57(12):1572–8.

4. Nagahara R, Mizutani M, Matsuo A, Kanehisa H, Fukunaga T. Association of Sprint Performance With Ground Reaction Forces During Acceleration and Maximal Speed Phases in a Single Sprint. Journal of Applied Biomechanics. 2018 Apr;34(2):104–10.

5. Welch N, Richter C, Moran K, Franklyn-Miller A. Principal Component Analysis of the Associations Between Kinetic Variables in Cutting and Jumping, and Cutting Performance Outcome. Journal of Strength and Conditioning Research [Internet]. 2019 Feb 6 [cited 2022 Dec 4];Publish Ahead of Print. Available from: https://journals.lww.com/00124278-900000000-94949

6. Spiteri T, Cochrane JL, Hart NH, Haff GG, Nimphius S. Effect of strength on plant foot kinetics and kinematics during a change of direction task. European Journal of Sport Science. 2013 Nov;13(6):646–52.

7. Havens KL, Sigward SM. Cutting Mechanics: Relation to Performance and Anterior Cruciate Ligament Injury Risk. Medicine & Science in Sports & Exercise. 2015 Apr;47(4):818–24.

8. McMahon JJ, Suchomel TJ, Lake JP, Comfort P. Understanding the Key Phases of the Countermovement Jump Force-Time Curve. Strength & Conditioning Journal. 2018 Aug;40(4):96–106.

9. Linthorne NP. Analysis of standing vertical jumps using a force platform. American Journal of Physics. 2001 Nov;69(11):1198–204.

10. Nagahara R, Naito H, Miyashiro K, Morin JB, Zushi K. Traditional and ankle-specific vertical jumps as strength-power indicators for maximal sprint acceleration. J Sports Med Phys Fitness. 2014 Dec;54(6):691–9.

11. Bosco C, Tarkka I, Komi P. Effect of Elastic Energy and Myoelectrical Potentiation of Triceps Surae During Stretch-Shortening Cycle Exercise. Int J Sports Med. 1982 Jun;03(03):137–40.
