# Supplementary material for: Effects of a forefoot strengthening protocol on explosive tasks performance and propulsion kinetics in athletes: a single-blind randomized controlled trial
Source: PLoS One. 2025 Jun 2;20(6):e0313979. doi: 10.1371/journal.pone.0313979 (PMC12129235; doi:10.1371/journal.pone.0313979)

Supplemental Table 1. Results of linear mixed model and individual responses comparing groups (TRAINING versus CONTROL) after post-training and post-detraining period for absolute metatarsophalangeal joints maximal isometric flexion torque

		BASELINE	POST-TRAINING					POST-DETRAINING				
Variable	Group	S1 (week 1) & S2 (week 5)	S3 (week 14)	S3 vs Baseline (Adjusted mean difference)		Between-Group differences	Individual responses	S4 (week 18)	S4 vs Baseline (Adjusted mean difference)		Between-Group differences	Individual responses
		Mean ±SD	*Mean ±SD*	*Δ ± [95% CI]*	*ES + [95% CI]*	*ES + [95% CI]*	*Pos/ Triv/ Neg (% pos)*	*Mean ± SD*	*Δ ± [95% CI]*	*ES + [95% CI]*	*ES + [95% CI]*	*Pos/ Triv/ Neg (% pos)*
**DOMINANT FOOT**												
Absolute pushing force (N)	Training	268.7 ±90.1	355.2 ±82.0	**79.6 [63.4; 95.8]*** **	**1.82 [1.45; 2.19]**	**1.46 [1.09; 1.83]*** **	**11/1/0 (92%)**	350.3 ±87.3	**74.8 [58.6; 91.0]*** **	**1.71 [1.34; 2.08]**	**1.41 [1.04; 1.78]*** **	**11/1/0 (92%)**
	Control	260.0 ±53.0	259.8 ±64.2	0.1 [-15.3; 15.6]	0.00 [-0.37; 0.37]		1/11/1 (8%)	258.6 ±52.6	-1.4 [-16.5; 13.6]	0.01 [-0.41; 0.34]		1/11/2 (7%)
Absolute gripping force (N)	Training	88.8 ±38.8	106.5 ±51.1	**17.9 [4.2; 31.6]***	**0.48 [0.11; 0.85]**	0.24 [-0.16; 0.62]	1/11/0 (8%)	106.9 ±40.8	**18.3 [4.6; 32.1]***	**0.49 [0.12; 0.86]**	0.30 [-0.08; 0.67]	0/12/0 (0%)
	Control	75.1 ±24.9	85.4 ±28.3	7.9 [-5.3; 21.1]	0.22 [-0.15; 0.59]		0/13/0 (0%)	81.6 ±27.3	6.5 [-6.3; 19.3]	0.19 [-0.18; 0.56]		0/14/0 (0%)
Absolute total force (N)	Training	279.8 ±91.8	366.5 ±86.6	**79.7 [64.0; 95.3]*** **	**1.89 [1.52; 2.26]**	**1.45 [1.08; 1.82]*** **	**11/1/0 (92%)**	362.5 ±84.6	**75.6 [60.0; 91.3]*** **	**1.79 [1.42; 2.16]**	**1.45 [1.08; 1.82]*** **	**11/1/0 (92%)**
	Control	266.5 ±51.9	270.3 ±62.4	3.7 [-11.3; 18.6]	0.09 [-0.28; 0.46]		1/11/1 (8%)	266.9 ±52.3	0.4 [-14.1; 14.9]	0.01 [-0.36; 0.38]		1/11/2 (7%)
Relative total torque (Nm/kg)	Training	0.26 ±0.08	0.35 ±0.07	**0.07 [0.06; 0.09]*** **	**1.80 [1.42; 2.15]**	**1.36 [1.00; 1.74]*** **	**11/1/0 (92%)**	0.34 ±0.07	**0.07 [0.06; 0.09]*** **	**1.70 [1.35; 2.08]**	**1.34 [0.98; 1.72]*** **	**11/1/0 (92%)**
	Control	0.26 ±0.05	0.26 ±0.05	0.00 [-0.01; 0.02]	0.07 [-0.28; 0.43]		1/11/1 (8%)	0.26 ±0.05	0.00 [-0.01; 0.02]	0.01[-0.33; 0.35]		1/11/2 (7%)
**NON-DOMINANT FOOT**												
Absolute pushing force (N)	Training	247.7 ±85.1	331.9 ±80.8	**80.6 [65.7; 95.5]*** **	**2.01 [1.64; 2.38]**	**1.86 [1.49; 2.23]*** **	**11/1/0 (92%)**	327.1 ±87.6	**75.8 [61.0; 90.7]*** **	**1.89 [1.52; 2.26]**	**1.56 [1.19; 1.93]*** **	**11/1/0 (92%)**
	Control	244.1 ±66.7	237.2 ±67.8	-7.2 [-21.2; 7.1]	-0.18 [-0.55; 0.19]		0/11/2 (0%)	247.1 ±66.5	3.0 [-10.8; 16.8]	0.08 [-0.29; 0.45]		1/11/2 (7%)
Absolute gripping force (N)	Training	81.5 ±33.8	98.3 ±33.6	**17.8 [7.9; 27.7]** **	**0.67 [0.30; 1.04]**	**0.60 [0.23; 0.97]** **	1/11/0 (8%)	106.3 ±36.3	**25.7 [15.8; 35.6]*** **	**0.96 [0.59; 1.33]**	**0.95 [0.58; 1.32]*** **	3/9/0 (25%)
	Control	80.4 ±18.7	79.3 ±15.4	-1.2 [-10.7; 8.3]	-0.05 [-0.42; 0.32]		0/13/0 (0%)	76.6 ±23.7	-3.8 [-13.0; 5.4]	-0.15 [-0.52; 0.22]		0/13/1 (0%)
Absolute total force (N)	Training	256.6 ±87.9	339.2 ±85.0	**79.4 [64.1; 94.7]*** **	**1.92 [1.55; 2.29]**	**1.75 [1.38; 2.12]*** **	**11/1/0 (92%)**	339.2 ±90.4	**79.4 [64.1; 94.7]*** **	**1.92 [1.55; 2.29]**	**1.58 [1.21; 1.95]*** **	**11/1/0 (92%)**
	Control	252.2 ±65.4	246.2 ±65.9	-6.3 [-20.9; 8.3]	-0.16 [-0.53; 0.21]		0/11/2 (0%)	254.7 ±66.4	2.5 [-11.7; 16.7]	0.07 [-0.31; 0.44]		1/11/2 (7%)

*ES, Effect Size; Pos, Positive; Triv, Trivial; Neg, Negative; *p < 0.05; **p < 0.01; ***p<0.001*

Supplemental Table 2. Results of linear mixed model and individual responses comparing groups (TRAINING versus CONTROL) after post-training and post-detraining period for foot posture, foot morphology, quadriceps and ankle plantar flexors isometric torque

		BASELINE	POST-TRAINING					POST-DETRAINING				
Variable	Group	S1 (week 1) & S2 (week 5)	S3 (week 14)	S3 vs Baseline (Adjusted mean difference)		Between-Group differences	Individual responses	S4 (week 18)	S4 vs Baseline (Adjusted mean difference)		Between-Group differences	Individual responses
		Mean ±SD	*Mean ±SD*	*Δ ± [95% CI]*	*ES + [95% CI]*	*ES + [95% CI]*	*Pos/ Triv/ Neg (% pos)*	*Mean ± SD*	*Δ ± [95% CI]*	*ES + [95% CI]*		*Pos/ Triv/ Neg (% pos)*
**DOMINANT FOOT**												
Foot posture index score (AU)	Training	2.3 ±2.7	2.0 ±2.9	0.2 [-0.4; 0.7]	0.11 [-0.26; 0.48]	0.12 [-0.25; 0.49]	0/11/1 (0%)	1.9 ±2.6	0.1 [-0.4; 0.6]	0.05 [-0.32; 0.42]	0.24 [-0.13; 0.61]	1/11/0 (8%)
	Control	3.1 ±3.4	2.6 ±3.4	-0.9 [-0.6; 0.4]	-0.07 [-0.44; 0.30]		2/10/1 (15%)	2.9 ±3.5	-0.3 [-0.8; 0.2]	-0.23 [-0.60; 0.15]		3/10/1 (21%)
Navicular drop (mm)	Training	5.4 ±1.3	4.3 ±2.1	-1.1 [-2.6; 0.5]	-0.25 [-0.62; 0.12]	-0.29 [-0.66; -0.08]	1/11/0 (8%)	4.2 ±2.8	-1.2 [-2.7; 0.4]	-0.27 [-0.64; 0.10]	**-0.52 [-0.90; -0.15]***	0/11/1 (0%)
	Control	4.6 ±2.5	4.9 ±2.2	0.2 [-1.3; 1.8]	0.06 [-0.31; 0.43]		0/13/0 (0%)	5.7 ±2.4	1.2 [-0.4; 2.6]	0.28 [-0.09; 0.65]		0/13/1 (7%)
Arch height flexibility (mm/kN)	Training	7.5 ±3.5	7.7 ±3.5	0.1 [-2.3; 2.6]	0.02 [-0.35; 0.39]	-0.00 [-0.37; 0.37]	0/12/0 (0%)	6.4 ±4.2	-1.1 [-3.6; 1.3]	-0.17 [-0.54; 0.20]	-0.06 [-0.43; 0.31]	2/10/0 (17%)
	Control	8.3 ±3.1	8.4 ±3.2	0.1 [-2.3; 2.5]	0.02 [-0.35; 0.39]		0/13/0 (0%)	7.6 ±3.8	-0.7 [-3.0; 1.6]	-0.11 [-0.48; 0.26]		1/13/0 (7%)
Foot mobility magnitude (cm)	Training	0.63 ±0.16	0.63 ±0.23	0.00 [-0.13; 0.14]	0.01 [-0.36; 0.38]	-0.10 [-0.47; 0.28]	1/11/0 (8%)	0.61 ±0.20	-0.02 [-0.15; 0.12]	-0.05 [-0.42; 0.33]	-0.07 [-0.44; 0.30]	1/11/0 (8%)
	Control	0.70 ±0.18	0.73 ±0.29	0.04 [-0.01; 0.17]	0.10 [-0.27; 0.48]		0/13/0 (0%)	0.72 ±0.20	0.02 [-0.11; 0.15]	0.07 [-0.30; 0.44]		0/14/0 (0%)
Quadriceps isom. torque (Nm/kg)	Training	3.4 ±0.6	3.5 ±0.6	0.1 [-0.1; 0.2]	0.18 [-0.19; 0.55]	0.20 [-0.17; 0.57]	0/12/0 (0%)	3.5 ±0.6	0.0 [-0.0; 0.3]	0.19 [-0.18; 0.56]	0.10 [-0.27; 0.47]	1/11/0 (8%)
	Control	3.4 ±0.6	3.5 ±0.7	-0.0 [-0.2; 0.1]	-0.05 [-0.42; 0.32]		0/13/0 (0%)	3.5 ±0.8	0.0 [-0.1; 0.2]	0.08 [-0.29; 0.45]		0/14/0 (0%)
Ankle PF isom. torque (Nm/kg)	Training	1.7 ±0.3	1.8 ±0.3	0.0 [-0.1; 0.1]	0.03 [-0.34; 0.40]	0.03 [-0.33; 0.40]	0/12/0 (0%)	1.7 ±0.3	-0.1 [-0.2; 0.0]	-0.03 [-0.40; 0.34]	0.28 [-0.09; 0.64]	0/12/0 (0%)
	Control	1.7 ±0.4	1.7 ±0.4	-0.0 [-0.1; 0.1]	-0.05 [-0.41; 0.32]		0/13/0 (0%)	1.6 ±0.3	-0.0 [-0.1; 0.1]	-0.34 [-0.70; 0.03]		0/13/0 (0%)
**NON-DOMINANT FOOT**												
Foot posture index score (AU)	Training	3.3 ±4.2	3.3 ±4.2	0.1 [-0.9; 1.1]	0.03 [-0.34; 0.40]	0.05 [-0.32; 0.42]	0/12/0 (0%)	3.8 ±3.8	0.7 [-0.3; 1.7]	0.24 [-0.13; 0.61]	0.27 [-0.10; 0.64]	0/11/1 (0%)
	Control	3.7 ±2.8	4.1 ±3.7	0.6 [-0.4; 1.5]	0.22 [-0.15; 0.59]		0/12/1 (0%)	3.7 ±3.0	0.0 [-0.9; 0.9]	0.00 [-0.37; 0.37]		0/14/0 (0%)
Navicular drop (mm)	Training	6.2 ±2.0	5.9 ±2.3	-0.3 [-2.0; 1.4]	-0.07 [-0.44; 0.30]	-0.23 [-0.60; 0.14]	1/11/0 (8%)	5.1 ±2.0	-1.3 [-2.8; 0.6]	-0.25 [-0.62; 0.12]	-0.27 [-0.57; 0.17]	1/11/0 (8%)
	Control	4.8 ±3.2	4.8 ±3.1	-0.0 [-1.7; 1.6]	0.00 [-0.37; 0.37]		2/10/1 (15%)	4.6 ±2.7	-0.2 [-1.8; 1.4]	-0.05 [-0.42; 0.33]		2/12/0 (14%)
Arch height flexibility (mm/kN)	Training	8.2 ±2.6	9.2 ±3.5	0.1 [-1.1; 3.1]	0.17 [-0.20; 0.54]	0.33 [-0.04; 0.70]	0/12/0 (0%)	8.9 ±2.6	0.7 [-1.4; 2.8]	0.13 [-0.24; 0.50]	-0.04 [-0.41; 0.33]	0/11/1 (0%)
	Control	7.8 ±2.2	6.4 ±5.3	-1.3 [-3.4; 0.7]	-0.24 [-0.61; 0.01]		2/11/0 (15%)	8.8 ±3.8	1.0 [-1.0; 3.0]	0.19 [-0.18; 0.56]		0/13/1 (0%)
Foot mobility magnitude (cm)	Training	0.63 ±0.18	0.74 ±0.21	0.12 [0.00; 0.23]	0.37 [0.00; 0.75]	0.14 [-0.23; 0.52]	0/11/1 (0%)	0.69 ±0.19	0.07 [-0.05; 0.18]	0.22 [-0.15; 0.60]	-0.06 [-0.43; 0.31]	1/10/1 (8%)
	Control	0.65 ±0.13	0.68 ±0.20	0.03 [-0.08; 0.14]	0.10 [-0.28; 0.47]		1/12/0 (8%)	0.75 ±0.26	0.10 [-0.02; 0.21]	0.32[-0.05; 0.69]		0/13/1 (0%)

*AU, Arbitrary Unit; ES, Effect Size; Pos, Positive; Triv, Trivial; Neg, Negative; Isom., Isometric; PF, Plantar flexors; *p < 0.05.*

Supplemental Table 3. Secondary results of linear mixed model and individual responses comparing groups (TRAINING versus CONTROL) after post-training and post-detraining period for overall performance during sprinting and jumping

		BASELINE	POST-TRAINING					POST-DETRAINING				
Variable	Group	S1 (week 1) & S2 (week 5)	S3 (week 14)	S3 vs Baseline (Adjusted mean difference)		Between-Group differences	Individual responses	S4 (week 18)	S4 vs Baseline (Adjusted mean difference)		Between-Group differences	Individual responses
		Mean ±SD	*Mean ±SD*	*Δ ± [95% CI]*	*ES + [95% CI]*	*ES + [95% CI]*	*Pos/ Triv/ Neg (% pos)*	*Mean ± SD*	*Δ ± [95% CI]*	*ES + [95% CI]*	*ES + [95% CI]*	*Pos/ Triv/ Neg (% pos)*
**OVERALL PERFORMANCE**												
10-m sprint time (s)	Training	2.01 ±0.16	1.95 ±0.07	-0.05 [-0.06; -0.00]	-0.40 [-0.78; -0.03]	-0.25 [-0.62; 0.63]	3/9/0 (25%)	1.94 ±0.10	**-0.06 [-0.11; -0.01]***	**-0.48 [-0.86; -0.11]**	-0.26 [-0.63; 0.10]	**4/8/0 (33%)**
	Control	2.00 ±0.09	2.00 ±0.13	-0.01 [-0.06; 0.03]	-0.11 [-0.48; 0.27]		0/13/0 (0%)	1.98 ±0.13	-0.02 [-0.07; 0.02]	-0.17 [-0.53; 0.20]		1/12/1 (7%)
17-m sprint time (s)	Training	2.93 ±0.25	2.86 ±0.17	-0.06 [-0,11; -0.01]	-0.42 [-0.79;-0.04]	-0.30 [-0.67; 0.08]	2/10/0 (17%)	2.89 ±0.20	-0.02 [-0,07; -0.03]	-0.17 [-0.54; 0.20]	-0.14 [-0.51; 0.23]	0/10/1 (0%)
	Control	2.91 ±0.16	2.92 ±0.19	-0.01 [-0.10; 0.08]	-0.07 [-0.43; 0.29]		1/11/1 (8%)	2.92 ±0.18	0.01 [-0.35; 0.38]	0.00 [-0.37; 0.38]		1/10/3 (7%)
34-m sprint time (s)	Training	4.72 ±0.21	4.69 ±0.29	-0.03 [-0,14; 0.07]	-0.19 [-0.81; 0.43]	-0.11 [-0.73; 0.51]	1/2/1 (25%)	4.68 ±0.29	-0.04 [-0,15; 0.06]	-0.25 [-0.87; 0.37]	-0.39 [-1.01; 0.23]	1/3/0 (25%)
	Control	4.95 ±0.23	4.95 ±0.24	-0.01 [-0.10; 0.08]	-0.08 [-0.70; 0.54]		0/4/1 (0%)	4.98 ±0.23	0.04 [-0.05; 0.12]	0.27 [-0.34; 0.89]		0/5/1 (0%)
Vertical CMJ jump height (cm)	Training	34.0 ±7.8	36.0 ±6.9	**1.3 [0.5; 2.0]****	**0.63 [0.26; 1.00]**	**0.71 [0.34; 1.08]*****	0/12/0 (0%)	35.2 ±7.1	0.5 [-0.3; 1.2]	0.24 [-0.13; 0.61]	**0.44 [0.06; 0.81]***	0/12/0 (0%)
	Control	32.9 ±6.9	32.5 ±7.2	-0.4 [-1.1; 0.3]	-0.20 [-0.57; 0.17]		0/13/0 (0%)	32.4 ±7.0	-0.5 [-1.2; 0.2]	-0.26 [-0.63; 0.11]		0/14/0 (0%)
FARJ reactive strength ratio (AU)	Training	1.29 ±0.38	1.45 ±0.42	0.12 [0.02; 0.22]	0.44 [0.07; 0.80]	**0.44 [0.07; 0.82]***	2/10/0 (17%)	1.42 ±0.36	0.09 [-0.01; 0.19]	0.33 [-0.04; 0.70]	**0.70 [0.33; 1.06]*****	2/10/0 (17%)
	Control	1.28 ±0.25	1.27 ±0.30	-0.01 [-0.10; 0.09]	-0.03 [-0.40; 0.33]		1/12/0 (8%)	1.16 ±0.28	-0.12 [-0.21; -0.02]	-0.47 [-0.84; -0.10]		0/12/2 (0%)

*CMJ, Countermovement Jump; FARJ, Foot-Ankle Rebound Jump; AU, Arbitrary Unit; ES, Effect Size; Pos, Positive; Triv, Trivial; Neg, Negative; *p < 0.05; **p < 0.01; ***p<0.001*

Supplemental Figure 1. Group average and individual participant data displayed in time-series graphs and individual mean differences changes in comparison to the minimal detectable changes (MDC) post-training and detraining period. Subgraphs A&B) 10-m sprint time (s); C&D) 17-m sprint time (s) and E&F) 34-m sprint time.

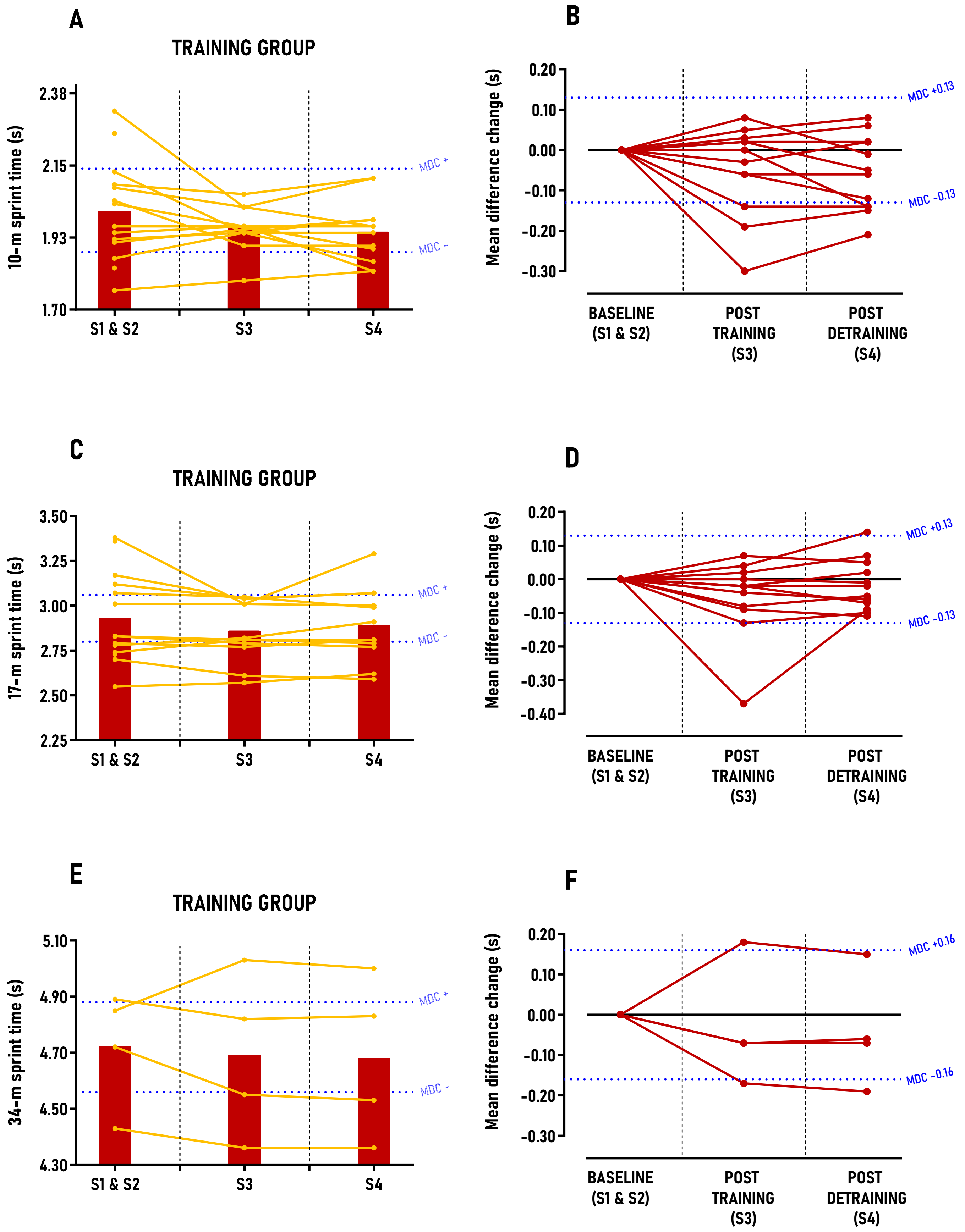

<u>Supplemental Figure 2</u>. Group average and individual participant data displayed in time-series graphs and individual mean differences changes in comparison to the minimal detectable changes (MDC) post-training and detraining period. Subgraphs A&B) Vertical countermovement jump (CMJ) jump height (cm); C&D) Foot-ankle rebound jump test - reactive strength ratio (AU) and E&F) Concentric RF – horizontal CMJ (%).

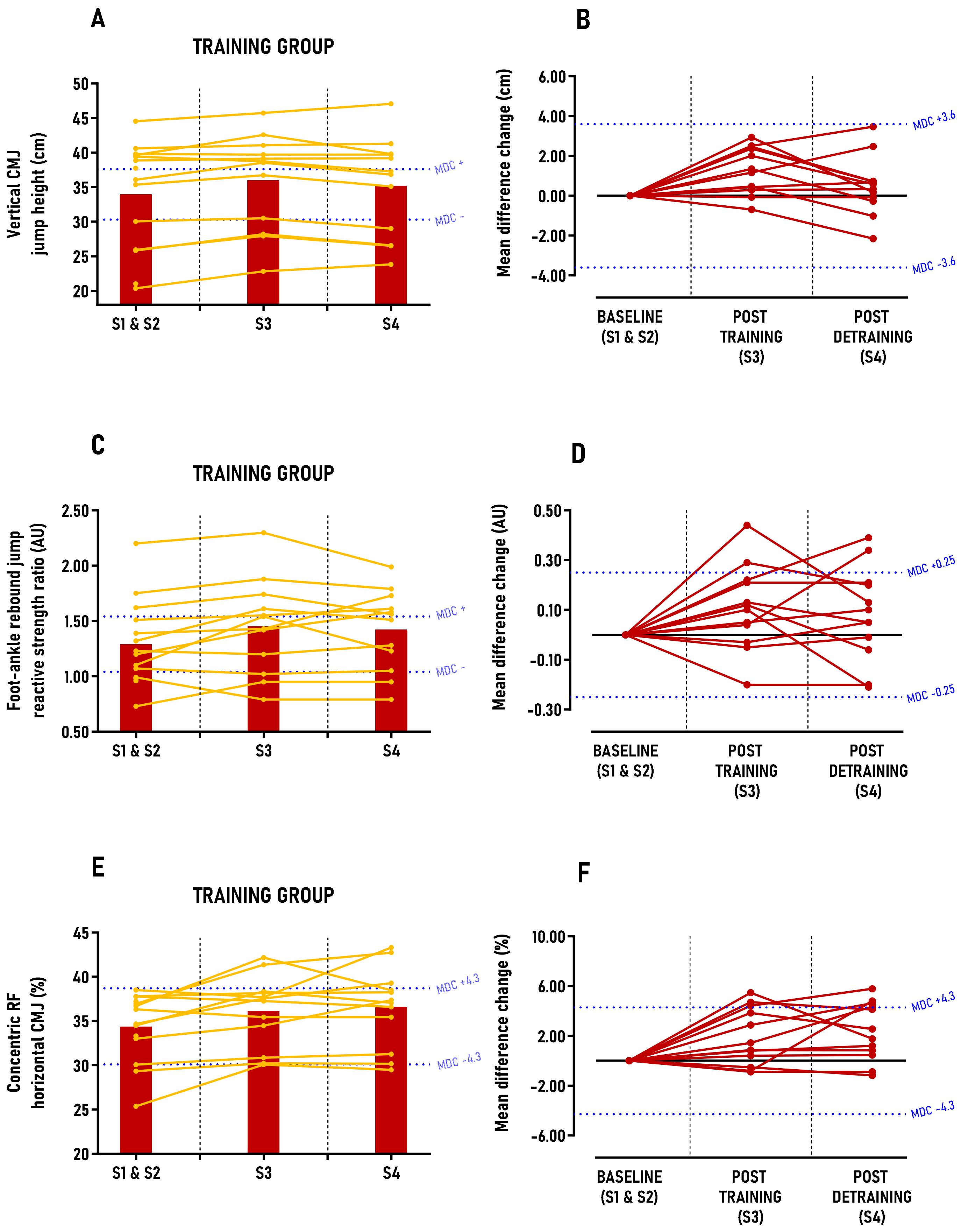

Supplement: S4 File — (PDF) [file pone.0313979.s004.pdf]
