# Supplementary material for: Effects of a forefoot strengthening protocol on explosive tasks performance and propulsion kinetics in athletes: a single-blind randomized controlled trial
Source: PLoS One. 2025 Jun 2;20(6):e0313979. doi: 10.1371/journal.pone.0313979 (PMC12129235; doi:10.1371/journal.pone.0313979)
Supplement: Attachment — Submitted filename: Authors response to reviewer 2.docx [file pone.0313979.s006.docx]

**Response to Reviewer 2**

Date: March, 2025

| Reviewer 2 |  |  |
| --- | --- | --- |
| *Comments* | *Answers/Corrections* | *Line* |
| General comments | | |
| The present work represents an extremely interesting and many times underestimated topic.  The foot muscles represent a factor that should always be taken into account when explosive tasks such as those analyzed in this study are required. The proposed training methodology and its strength principles represent a significant strength.  The manuscript is well-written, well-structured, with a consistent hypotesis and useful conclusion. The amount of additional material provided is rich in information and helps the reader learn more about the methodology used. I have only a few clarification to ask. | We thank the reviewer for his/her appreciated comment on our manuscript and we did our best to enhance the understanding of the article by addressing each comment. |  |
| *Specific comments* | | |
| 1. In line 83, clarifying or expanding the meaning of “training is testing protocol” might help in reading the manuscript. | Corrected with: “Moreover, most studies included moderately trained participants; with strengthening exercises and strength evaluation performed on the same ergometer; this led to a potential bias toward greatest strength and athletic performance improvement” | Lines 82-5 |
| 2. In the supplemental content (S3 Study Protocol Design), the height of the timing gates is 1.20m. For what reason was this height selected? In a three-point crouched start position, could the variation of the body during a sprint lead to measurement errors? | This height was selected based on previous biomechanical studies using this height (as is it close to the body’ center of mass):  Marshall (2014): <https://pubmed.ncbi.nlm.nih.gov/24662232/>  Welch (2019):  <https://pubmed.ncbi.nlm.nih.gov/30664108/>  <https://pubmed.ncbi.nlm.nih.gov/30741857/>  The three-point crouched start position was used for standardization and the timing gates were placed 1m after the starting point to avoid potential sprint measurement errors. | S3 |
